# Lymph node Yield in Apical Tissue During Triangle Operation While Doing Whipples Operation: An Observational Study

**DOI:** 10.31729/jnma.8887

**Published:** 2025-02-28

**Authors:** Dhiresh Kumar Maharjan, Prashanta Pudasaini, Bidur Prasad Acharya, Yugal Limbu, Roshan Ghimire, Prabin Bikram Thapa

**Affiliations:** 1 Department of Gastrointestinal and General Surgery, Kathmandu Medical College Teaching Hospital, Sinamangal, Kathmandu, Nepal

**Keywords:** *apical tissue*, *Triangle operation*, *Whipple's operation*

## Abstract

**Introduction:**

The "TRIANGLE operation" involves the en-bloc removal of the tumor and the entire "mesopancreas" from the triangle-shaped space bounded by the superior mesenteric artery, coeliac trunk, and portal vein. This study assessed lymph node yield in apical tissue during the triangle operation.

**Methods:**

An observational cross-section study was conducted for two years at the Department of Gastrointestinal and General Surgery at a tertiary care center of Nepal. from 15th March 2022 to 15th March 2024. The operative procedure included pancreaticoduodenectomy or Whipple's operation. Total sampling was done. Postoperative outcome and lymph node yeild of the surgeries were studied. Ethical approval was taken from the Institutional Review Committee (Reference No-1102202204).

**Results:**

A total of 56 patients underwent pancreaticoduodenectomy along with a triangle operation. The mean age of the patients was 56.38±14.79 years. Male: Female ratio was 1.2:1. Mean preoperative BMI was 24.41±4.72. The mean total lymph node yield was 20.95±8.57. Nineteen patients had a positive lymph node yield in the triangle tissue. The mean triangle lymph node yield was 10.59±4.92, and the mean positive lymph node was 2.58±1.64. Among 56 patients, nine patients had both apical margin and the rest of the triangle tissue margin positive. Ten patients had negative apical tissue margins, but the rest of the triangle tissue was positive, whereas, in thirty-seven patients, both the apical tissue and the rest of the triangle circumferential resection margin tissue were negative.

**Conclusions:**

This study emphasizes the importance of the inclusion of apical tissue dissection at the confluence of SMA and coeliac trunk to achieve R0 resection. However, a long-term follow is awaited.

## INTRODUCTION

Pancreaticoduodenectomy or Whipple's operation has been standard operative management for periampullary carcinoma including pancreatic ductal adenocarcinoma and ampullary malignancy.^1-4^ It has been associated with high morbidity ranging from 20 to 59%, mostly because of postoperative pancreatic fistula (POPF) 5 to 16 %,delayed gastric emptying (DGE) 10 to 45%^5-8^ and post pancreatic hemorrhage (PPH) 10 to 38%,^9-12^ along with 30-day mortality rate of 0.9% to 8.1% in high-volume centers.^13-16^

Despite overall 5-year survival for pancreatic ductal carcinoma being 18 to 22 %, better results of 5-year survival have been shown by the Heidelberg group up to 54% for early Tis-T2.^2^ This improvement has been attributed to the improvement of surgical approach "triangle operation".

However, the apex of the triangle is the ultimate basin of drainage of lymph from around the pancreatic head region, hence, it should be described as a separate entity with importance.

Hence, this study aims to assess the lymph node yield in apical tissue while doing triangle surgery, look for a positive lymph node ratio among the triangle tissue, and correlate with duration of operation, postoperative complications, and pathological TNM staging.

## METHODS

This is a descriptive observational study conducted at the Department of Gastrointestinal Surgery and General Surgery, at Kathmandu Medical College & Teaching Hospital, between 15^th^ March 2022 to 15^th^ March 2024.

All patients over the age of 18yearswhowere admitted to the surgical unit with the diagnosis of periampullary and ampullary carcinoma, carcinoma head of pancreas who underwent pancreaticoduodenectomy with resectable and borderline resectability status as described according to consensus statement by International Study Group of Pancreatic Surgery (ISGPS)^5^. Borderline resectable pancreatic adenocarcinoma (Borderline PDAC) received preoperative neoadjuvant chemotherapy (Gemcitabine 1000mg/m^2^ + nab- paclitaxel 125mg/m^2^ on days 1,8 and 15 of each 28-day cycle) for three cycles and repeat CT scan after three cycles to assess tumor progression or regression. The patients who had double primary, metastatic disease and those who didn't consented to the study were excluded from the study. An ethical approval was taken from the Institutional Review Committee (Reference No-1102202204). All patient meeting the inclusion criteria during the study period were included in the study. Total population sampling was done. Baseline parameters included age, sex, body mass index (BMI) and the American Society of Anesthesiologist's classification (ASA) were noted.^6^ Clavian-Dindo classification was used to classify postoperative morbidity.^7^

The primary outcomes of the study include lymph node yield during resection, the presence of positive lymph nodes, and the lymph node ratio. The secondary outcomes comprise the duration of the operation, postoperative complications (if available, classified according to the Clavien-Dindo classification), and the pathological TNM stage.

While the primary focus was on assessing lymph node yield, other operative parameters were also investigated to examine the implications of extensive dissection around the triangle and its apex. Operative parameters included operation time, blood loss, the need for transfusion, and details of the surgical procedure.

All patients underwent staging laparoscopy to rule out peritoneal or hepatic metastases, followed by laparotomy and the initial steps of the operation included the Whipple's procedure with SMA margin. In this above method, though most of the soft tissue within the triangle were dissected out, apical tissue clearance at the origin of the coeliac trunk and SMA were uncertain when described according to the triangle dissection by the Heidelberg technique.^8^

In this study, we have labeled the apical tissue of the triangle as a separate entity that consists of periarterial neurolymphatic tissues which are present at the apex of the triangle formed by the origin of the coeliac trunk and SMA. We marked the edge of the apical tissue separately with methylene blue for proper orientation of the specimen to look for circumferential resection margin (CRM).

The data were analyzed using IBM SPSS Statistics for Windows, version 16 (IBM Corp., Armonk, N.Y., USA). Categorical values were expressed in mean and standard deviation and percentage.

## RESULTS

Total of 56 patients underwent the Whipple's operation during this study period. The average age of the patients was 56.38±14.79 years. Male: Female ratio was 1.2:1. Mean preoperative BMI was 24.41±4.72. ASA grading 2 and 3 were present in 17(30.35%) and 3(5.35%) patients respectively. Previous common bile duct (CBD) interventions included percutaneous trans-hepatic biliary drainage (PTBD) in 10(17.85%) patients and endoscopic retrograde cholangiopancreatography (ERCP) in 4(7.14%) patients. Comorbidities such as hypertension, diabetes mellitus and cardiopulmonary dysfunction were present in 27(48.21 %) patients (Table 1).

**Table 1:** Demographic of patients undergoing TRIANGLE Operation (n=56).

**Patient demographics**	
Age (Mean±SD)	56.38±14.79
Gender (M;F)	1.2:1
BMI (kg/m2)	24.41± 4.72
ASA Grading	n (%)
ASA 1	36(64.28)
ASA 2	17 (43.58)
ASA 3	3 (7.69)
**Operation Performed**	
Pancreaticoduodenectomy	56 (100)
Resectable	42(75)
Borderline resectable (post neoadjuvant)	14(25)
Indication of pancreaticoduodenectomy	
Distal Cholangiocarcinoma	22 (39.28)
Ampullary Adenocarcinoma	14 (25)
Pancreatic head Adenocarcinoma	20 (35.71)
**Previous Interventions**	
PTBD	10 (17.85)
ERCP	4 (7.14)
**Previous Co-morbidities**	
Diabetes Mellitus	16 (28.57)
Hypertension	6 (10.71)
Cardiopulmonary Dysfunction	5 (8.92)

PTBD: Percutaneous trans-hepatic biliary drainage; ERCP: Endoscopic retrograde cholangiopancreatography

Amongst these patients, the total LN yield was 20.95 ± 8.57 and the triangle LN yield was 10.59 ± 4.92. The mean operating time was 325.41±59.62 min with an average blood loss of 442.75±130.72 ml. Average hospital stay 8.92± 2.8 days (Table 2 and Table 3).

**Table 2:** Operative parameters of the patients undergoing TRIANGLE operation (n=56).

Characteristics	
Operation time (Mean±SD)	325.41±59.62 min
Blood loss (Mean±SD)	442.75±130.72 ml
**Pathological staging**	
T2N0	5 (44.64)
T2N1	21 (37.50)
T2N2	20 (7.14)
T3N1	4 (7.14)
T3N2	6(3.57)
**Clavian Dindo Classifications:**	
Grade I	8 (14.29%)
Grade II	14 (25.00%)
Grade IIIa	1 (1.79%)
Grade IIIb	2 (3.57%)
Grade IVa	2 (3.57%)
Grade IVb	1 (1.79%)
Grade V	2 (3.57%)
**Post-operative Complication**	
Postoperative pancreatitis	7 (12.5%)
DGE	10 (17.85%)
- DGE grade A	8 (14.28%)
- DGE grade B	2 (3.57%)
POFF	6 (10.71%)
- POPF grade B	5 (8.92%)
- POPF grade C	1 (1.78%)
Superficial wound site infections	5 (8.92%)
Chyle leak	3 (5.35%)
Pseudoaneurysm	2 (3.57%)
Postoperative diarrhea	6 (10.71%)
Death	2 (3.57%)
**Hospital stay (Mean±SD)**	8.92± 2.8 days

DGE: Delayed Gastric Emptying; POPF: Postoperative Pancreatic Fistula; T2N0, T2N1, T2N2, T3N1, T3N2: Tumor (T) and Node (N) staging based on pathological findings.

**Table 3:** Lymph Node(LN) yield in patients undergoing TRIANGLE Operation (n=56).

Lymph Node yield	Mean±SD
Total LN (TLN) yield	20.95±8.57
Triangle LN yield	10.59±4.92
Positive LN yield in triangle tissue	2.58 ±1.64

**Table 4:** Circumferential Resection Margin status of apex and rest of the triangle tissue (n=56).

	n (%)
Both Apical tissue and rest of triangle tissue Positive	9 (16.07)
Rest of Triangle tissue positive except Apex	10 (17.86)
Both apical tissue and rest of triangle tissue negative	37 (66.07)

Among 56 operated patients,9 (16.07%) patients had both apical margin and the rest of the triangle tissue margin positive, 10 (17.86 %) had negative apical tissue margins but the rest of the triangle tissue was positive, whereas in 37(66.07%) patients, both the apical tissue and the rest of the triangle circumferential resection margin tissue were negative (Table 4).

There were two postoperative mortality, one patient with persistent chyle leak with sepsis and septic shock on 28 days postoperative day and another one with grade C postoperative pancreatic fistula(POPF), reexplored on 7^th^ POD for completion pancreatectomy and splenectomy but death on 10^th^ POD with septic shock.

Thirty out of 56 patients (53.57%) experienced surgical morbidity. Complications were classified as Clavien-Dindo classification (Table 2) with grade IV a(2) , and grade IVb(1), among whom two patients eventually had mortality Grade V(2). Two patients with Clavien-Dindo grade 3b with pseudoaneurysms were managed with interventional radiological coiling.

## DISCUSSION

In our study, apex of the triangle which consists of periarterial neurolymphatic tissues was positive in nine out of 56 patient(16.07%) with a positive lymph node yield of 2.58±1.64. whereas the total triangle lymph node yield averaged 10.59±4.92. Notably, a considerable body of research supports an optimal cutoff range for LN yield in the range of 10-15 nodes.^9^ As exemplified by a study conducted by Huebner et al. an examination of 11 or more lymph nodes (TLN≥11) demonstrated an enhanced accuracy in survival prognosis for node-negative patients. Conversely, scrutinizing fewer than 11 lymph nodes (TLN 1-10) was associated with probabilities ranging from 10 % to 41 % of potentially overlooking one or more positive lymph nodes, contingent upon the actual number of nodes examined.^10^ Despite the ongoing debate, the extraction of lymph nodes holds significant practical relevance in delineating cancer staging and predicting patient prognosis.^10-12^

In the context of PD, complete excision of the total mesopancreas has been described with extensive dissection around the mesopancreatic root, including dissection within the vascular sheath of major vessels.^13-15^ This approach, exemplified by the Heidelberg triangle operation, aims to skeletonize the vessels, thereby enhancing the number of retrieved lymph nodes, reducing complication rates, and improving the efficacy of radical treatment.^8^

Even before the coining of the term "triangle operation," Igor Ignjatovic et al. observed that an extended lymphadenectomy during radical surgical treatment for pancreatic head carcinoma yielded more lymph nodes (24.1±6.5) compared to a standard pancreatoduodenectomy (18.5±5), and this difference was statistically highly significant.^18^ Our study similarly shows an increased lymph node yield (6-10 nodes) in the triangle tissue when assessed separately, underscoring the importance of dissecting and evaluating this tissue distinctly.

Though metanalysis of extended versus standard lymphadenectomy during pancreaticoduodenectomy done by Orci LA, in 2015 have showed prolonged operation duration mean difference 63 mins (95%CI 29-96;p=<0.001), increased need of blood transfusion mean difference 0.20 units and increased post operative morbidity(OR 1.5,95% CI 1.25-2.00;p=0.030).^19^ Our study have shown mean duration of operation was 325.41±59.62 min with range from 235 mins to 410 mins which was comparable to other study where extended lymphadenectomy have been shown 45mins to 63 mins more than standard lymphadenectomy .^19,20^

Circumferential dissection of nerve plexus around the Coeliac artery and superior mesenteric artery have been attributed intractable diarrhea leading to malnutrition and poor quality of life,^21,22^ however in our study only 10.71% of patient had diarrhea which was better after 3 months and was lesser than randomized controlled study done by Yang JY et from Korea with 15% in extended lymphadenectomy patients.^23^ However, chyle leak was observed in 3 patient (5.35%) among which one patient had severe persistent chyle leak leading to sepsis and death. Incidence of chyle leak have been reported from 1%-16 % during pancreaticoduodenectomy and have been associated more with extended lymphadenectomy leading to hypoalbuminemia, lymphocytopenia, and impaired wound healing.^24,25^

Though incidence of delayed gastric emptying ranges from 19-57% ^26-28^ and post operative pancreatic fistula(POPF) of 10-34% ^29^ after standard pancreaticoduodenectomy and triangle operation ,our study showed DGE of 17.85% while POPF of 10.71% among which one patient with grade C POPF underwent completion pancreatectomy but did not survive.

Numerous studies are underway, reshaping paradigms in pancreatic cancer treatment, one of which is the TRIANGLE trial. This multicenter randomized controlled superiority trial with two parallel study groups aims to assess, if a more radical Triangle dissection alongside conventional pancreaticoduodenectomy enhances Disease-Free Survival (DFS) and improves oncological outcomes for patients with pancreatic head carcinomas.^3^

Therefore, this study highlights the significance of apical tissue dissection in determining the lymph node yield and oncological outcomes during triangle surgery for pancreatic and peri-pancreatic cancer.

The small sample size is a limitation of this work, but it does demonstrate the significance of apical tissue as a distinct entity in triangle operation and warrants consideration for R0 resection. However, we are following up these patient for long term survival benefit of extensive dissection around triangle tissue and effect of lymph node dissection in terms of survival.

## CONCLUSIONS

This study emphasizes the significance of dissecting apical tissue around the SMA and celiac trunk to enhance lymph node yield while minimizing shortterm morbidity. Nonetheless, long-term follow-up is imperative to monitor tumor-free survival and recurrence.
